# Foreign Body Ingestion Followed by Appendiceal Perforation

**DOI:** 10.1155/2021/8877671

**Published:** 2021-03-29

**Authors:** Sarah Qassim, Ali Lairy, Sami Asfar

**Affiliations:** ^1^Department of Surgery, Mubarak Al-Kabeer Hospital, Kuwait; ^2^Kuwait University, Kuwait

## Abstract

**Background:**

Foreign body ingestion is very common among specific groups, especially children. However, appendicitis and perforated appendix caused by a foreign body is rare. *Case summary*. A 40-year-old female presented with abdominal pain in the right lower quadrant of 10 days duration after accidentally ingesting a drilling bit during a dental procedure. She had right iliac fossa tenderness on physical examination. X-ray showed a pointed long metal object in the right lower quadrant. A contrast-enhanced computed tomography scan of the abdomen revealed a pointed metal object in the pelvis with inconclusive location. Diagnostic laparoscopy showed an inflamed appendix with the tip of the metal object perforating it. Appendectomy was performed. Histopathology showed an inflamed appendix.

**Conclusion:**

Foreign bodies that cause appendicitis are rare. However, they may become lodged at any site of the gastrointestinal tract and cause inflammation or perforation. This is a bizarre case of foreign body-induced appendicitis with perforation.

## 1. Background

Acute appendicitis is a very common presentation, and it usually occurs between the extreme of ages. The pathophysiology of acute appendicitis is obstruction of the appendiceal lumen. The obstruction is either by fecal material, enlarged lymphoid follicle, or foreign material. Foreign body ingestion is very common among children and mentally disabled adults. Foreign bodies either pass spontaneously or need endoscopic removal. Surgical intervention is rarely required. It is diagnosed with X-ray and sometimes computerized tomography (CT) of the abdomen if perforation is suspected. Management depends on the timing and the type of the ingested material. This case report presents a rare case of perforated appendix that is due to foreign body ingestion, which is a drilling bit.

## 2. Case Presentation

**Figure 1:**
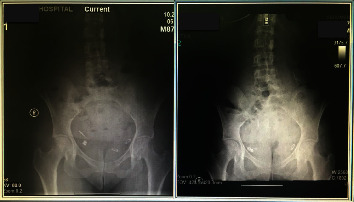
Two abdominal X-ray images of the patient. On the right, abdominal X-ray on first day of foreign body ingestion. On the left, 10 days after foreign body ingestion. Both images shows a right lower quadrant pointed metal object.

**Figure 2:**
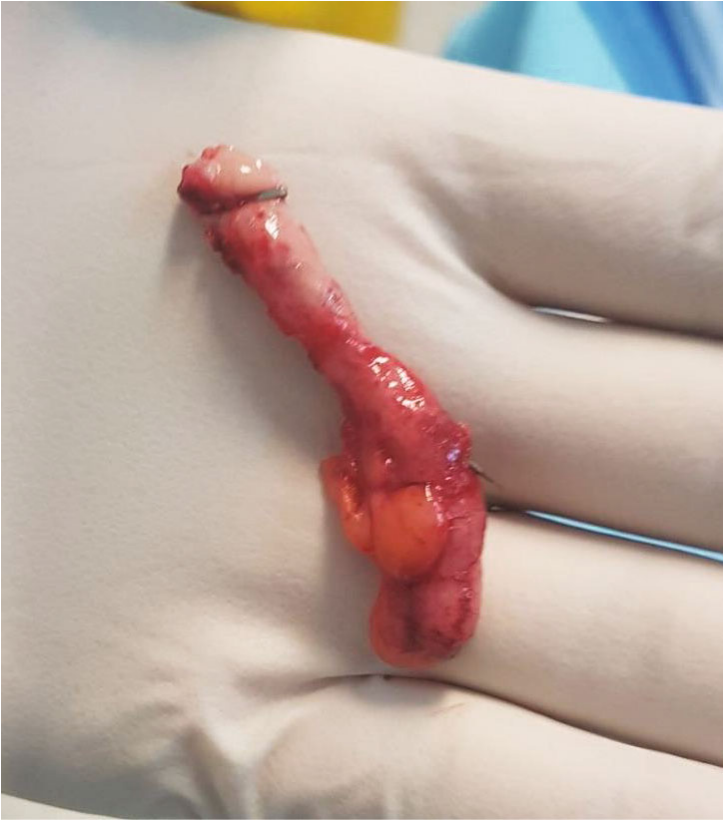
The inflamed appendix and the metal object penetrating from it.

**Figure 3:**
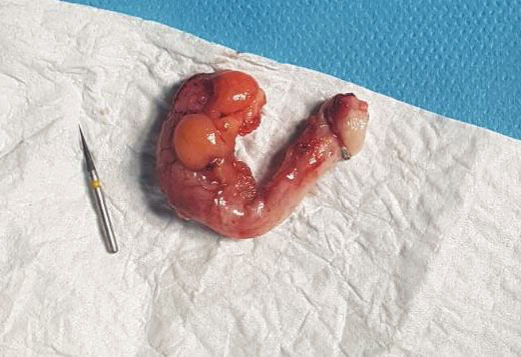
Inflamed appendix and the metal object.

A 40 years old lady presented with right iliac fossa pain of 10 days duration. The pain was intermitted at the beginning and then became severe within the last 3 days. There were no radiation and no aggravating or relieving factors. The patient gave a history of accidentally ingesting a metal object (drilling bit) during a dental procedure 10 days prior to presentation. There were few episodes of vomiting. However, no change in bowel habits or micturition. She has a previous history of laparoscopic sleeve gastrectomy, bilateral tubal ligation, and abdominoplasty. Her abdomen was tender in the right iliac fossa with guarding and rebound tenderness. The laboratory tests were within normal limits. Abdominal X-ray showed a long pointed metal object in the right lower quadrant (Figure 1). Computerized tomography (CT) of the abdomen showed a metallic foreign body at the right pelvic region lateral to the metallic clips of previous tubal ligation. The foreign body slightly moved between the pre- and postcontrast scans. Another metallic clips were seen at the presacral region and left adnexa. No localized collection or pneumoperitoneum was seen. The patient underwent a diagnostic laparoscopy. An intestinal or colonic perforation was suspected. An inflamed appendix was found, and a metal pointed object was perforating the appendix (Figures 2 and 3). Appendectomy was performed as usual. Postoperative course was uneventful. Histopathology of the appendix showed acute necrotizing appendicitis with periappendicitis.

## 3. Discussion

Foreign body ingestions are very common especially among children and mentally disabled. The majority of the ingested material passes through the alimentary tract spontaneously of around 80%. Less than 1% causes perforation or obstruction and requires surgery [1]. Impaction of a foreign body usually occurs at gastrointestinal acute angulations (e.g., duodenojejunal junction, iliocecal valve, and appendix) and narrowed areas (e.g., strictures, adhesions, previous gastrointestinal surgery, or anomaly) [2]. Most of the ingested objects are coins, magnets, and button batteries. The management of ingested material depends on the timing of ingestion, type, size, shape, and location of the object. Acute appendicitis is a common presentation, and it is usually due to lymphoid hypertrophy within the appendices lumen or fecal impaction. Inflammation of the appendix is rarely caused by foreign body impaction in its lumen as most of them pass spontaneously. Few case reports in the literature have documented such rare presentation, and the symptoms depend on the type and size of the ingested foreign body. Antonacci et al. [3] described a rare case of appendicitis that is caused by marrowbone ingestion. It remained asymptomatic for 15 years, and the specimen revealed a calcified fecaloma in the lumen of the appendix. Another case with a staple ingestion resulted in delayed presentation and the formation of calcified appendicolith [4]. These cases show that the foreign body can remain dormant for months or even years until presentation and calcifications demonstrate chronicity [4]. And as magnet ingestion is common in children, it can lead to perforated appendix and even an ileocaecal fistula [5]. On the opposite of blunt objects, ingesting sharp long materials causes perforation [6]. Accidental ingesting of a drilling bit is a rare phenomenon, and careful evaluation of the signs and symptoms prompts immediate management. A similar case was reported by Tsukamoto in which a reamer (a root canal instrument) migrated to the appendix incidentally and caused appendiceal perforation in an asymptomatic patient [7]. Our patient became symptomatic within days. Because imaging failed to identify the exact location of the sharp foreign body, laparoscopy was necessary. Should appendiceal location be identified, we would have planned appendectomy even in asymptomatic patient owing to respective risks of perforation and abscess of 70% and 31% [8]. In conclusion, foreign body ingestion is common but is a rare cause of appendicitis. The appendix is a place where a foreign body can become impacted. If a sharp object is impacted within the lumen of the appendix, there is a high risk of perforation. Appendectomy should be promptly performed.

## Data Availability

Previously reported case reports were used to support this study and are available and cited at relevant places within the text as references.
